# The Book World of Medicine and Science

**Published:** 1893-08-19

**Authors:** 


### The Book World of Medicine and Science.

### BOOK REVIEWS.

A Text-Book of Domestic Economy (complete) adapted for Use in Training Colleges, Schools, and Nursing Institutions; and as a Domestic Book of Health. By F. T. Paul, F.R.C.S., Surgeon to the Liverpool Royal Infirmary. With 175 illustrations. (London: Longmans, Green, and Co. 1893.)

This is a most admirable book, and we wish it could be in every household in the land, and its wise, practical teaching made the guide in all our homes. The subject is divided into two parts, which may roughly be called the body and the home. A brief description of the structure and functions of the human body is first given, and this leads on naturally to chapters devoted to the consideration of foods of all lands, their preservation and cooking, clothing, cleanliness, and work and rest. In part 2 the construction of a home is first discussed, and then follow articles on ventilation, heating, fuels, lighting, water supply, drainage, income and expenditure, and thrift. In the concluding chapters the causes and prevention of disease, disinfection, nursing, the tending of the sick are dealt with. The range of subjects covered, therefore, is very wide, and we admit the justice of the addition of the word "complete" to the title of the book. It is a great help to have so many and varied matters dealt with in a single manual. Mr. Paul's manner of dealing with them is truly admirable. He is precise, simple so that a child might understand him, thoroughly practical and homely. He doesn't preach some impossible ideal of life, but tells plain men and women how they can all make their lives happier and better by exercising common sense and moderate diligence and foresight. The book is well printed on good paper, the illustrations in the main are good, and we hope it will have a very large circulation, as it might then be the means of doing a great deal of good.

The Cyclists' Route Book for Great Britain and Ireland, with index map in five sections. By W. T. Spurrier. (Published by Iliffe and Sons. 1s.)

The little volume before us will be heartily welcomed by that largely-increasing class of persons who intend to spend their holiday exploring the scenery of our own country, and at the same time to secure a wholesome amount of physical exercise. It would probably be difficult to find a more pleasureable form of recreation both of body and of mind than is afforded by a cycle tour. The constant change of scene throws one's thoughts out of the old worn ruts, and supplies a store of pleasant memories to last till holiday-time comes round again. And at the same time the muscular work involved in getting about, serves to renovate a body whose claims may have been perforce neglected owing to pressure of business pursuits. It is to satisfy the needs of the country rover that Mr. Spurrier's little book has been designed, and on the whole admirably executed. In its general tenour it reminds one of the road-book published by the Cyclist Touring Club. But of this latter work only that part which deals with the South of England is as yet published, and in the interim which must elapse before its completion, the present route-book will serve a most useful end. The directions are clear, and the character of the roads is given quite as fully as one could expect in a book small

**THE BOOK WORLD OF MEDICINE AND SCIENCE**—*continued.*

enough to be carried in the pocket. Cyclists will also appreciate the short notes on those points of local interest in the routes, which are so often passed by, when one is travelling, through ignorance or inadvertence. The practical hints to the less-experienced tourist, though here and there, perhaps, savouring somewhat of the pulpit, are extremely sensible, and are those of a practised rider, so that on all grounds we can cordially recommend the book as thoroughly fulfilling the purpose for which it was written.

